# Detection of viral RNA in diverse body fluids in an SFTS patient with encephalopathy, gastrointestinal bleeding and pneumonia: a case report and literature review

**DOI:** 10.1186/s12879-020-05012-8

**Published:** 2020-04-15

**Authors:** Kazumasa Akagi, Taiga Miyazaki, Kazuhiro Oshima, Asuka Umemura, Satoshi Shimada, Kouichi Morita, Hiroaki Senju, Masato Tashiro, Takahiro Takazono, Tomomi Saijo, Shintaro Kurihara, Motohiro Sekino, Kazuko Yamamoto, Yoshifumi Imamura, Koichi Izumikawa, Katsunori Yanagihara, Akihiko Uda, Shigeru Morikawa, Tomoki Yoshikawa, Takeshi Kurosu, Masayuki Shimojima, Masayuki Saijo, Hiroshi Mukae

**Affiliations:** 1 Department of Respiratory Medicine, Nagasaki University Hospital, Nagasaki, Japan; 2 Present Address: Department of Respiratory Medicine, Nagasaki Prefecture Shimabara Hospital, Nagasaki, Japan; 3 Department of Infectious Diseases, Nagasaki University Graduate School of Biomedical Sciences, Nagasaki, Japan; 4 Present Address: Department of Internal Medicine, Goto Central Hospital, Nagasaki, Japan; 5 Present Address: Department of Respiratory Medicine, Sasebo City General Hospital, Sasebo, Japan; 6 Department of Virology, Institute of Tropical Medicine (NEKKEN), Nagasaki University, Nagasaki, Japan; 7 Nagasaki University Infection Control and Education Centre, Nagasaki University Hospital, Nagasaki, Japan; 8 Division of Intensive Care, Nagasaki University Hospital, Nagasaki, Japan; 9 Department of Laboratory Medicine, Nagasaki University Graduate School of Biomedical Sciences, Nagasaki, Japan; 10 Department of Veterinary Science, National Institute of Infectious Diseases, Tokyo, Japan; 11 Department of Virology I, National Institute of Infectious Diseases, Tokyo, Japan

**Keywords:** SFTS, Viremia, Encephalopathy, Pneumonia, Case report

## Abstract

**Background:**

Severe fever with thrombocytopenia syndrome (SFTS) is an emerging infectious disease that commonly has a lethal course caused by the tick-borne Huaiyangshan banyang virus [former SFTS virus (SFTSV)]. The viral load in various body fluids in SFTS patients and the best infection control measure for SFTS patients have not been fully established.

**Case presentation:**

A 79-year-old man was bitten by a tick while working in the bamboo grove in Nagasaki Prefecture in the southwest part of Japan. Due to the occurrence of impaired consciousness, he was referred to Nagasaki University Hospital for treatment. The serum sample tested positive for SFTSV-RNA in the genome amplification assay, and he was diagnosed with SFTS. Furthermore, SFTSV-RNA was detected from the tick that had bitten the patient. He was treated with multimodal therapy, including platelet transfusion, antimicrobials, antifungals, steroids, and continuous hemodiafiltration. His respiration was assisted with mechanical ventilation. On day 5, taking the day on which he was hospitalized as day 0, serum SFTSV-RNA levels reached a peak and then decreased. However, the cerebrospinal fluid collected on day 13 was positive for SFTSV-RNA. In addition, although serum SFTSV-RNA levels decreased below the detectable level on day 16, he was diagnosed with pneumonia with computed tomography. SFTSV-RNA was detected in the bronchoalveolar lavage fluid on day 21. By day 31, he recovered consciousness completely. The pneumonia improved by day 51, but SFTSV-RNA in the sputum remained positive for approximately 4 months after disease onset. Strict countermeasures against droplet/contact infection were continuously conducted.

**Conclusions:**

Even when SFTSV genome levels become undetectable in the serum of SFTS patients in the convalescent phase, the virus genome remains in body fluids and tissues. It may be possible that body fluids such as respiratory excretions become a source of infection to others; thus, careful infection control management is needed.

## Background

Severe fever with thrombocytopenia syndrome (SFTS), an infectious disease caused by the tick-borne Huaiyangshan banyangvirus [former SFTS virus (SFTSV)], *Banyangvirus* [former *Phlebovirus*] genus, *Phenuiviridae* (former *Buniyaviridae*) family [1]. SFTS is a viral hemorrhagic fever with a high case fatality rate. Patients with SFTS show symptoms such as a sudden fever, vomiting, diarrhea, stomachache, and general fatigue in the early phase of the disease, followed by hemorrhagic symptoms and unconsciousness in severe cases [2, 3]. Thrombocytopenia, leukopenia and elevation in liver enzymes are commonly demonstrated in blood examination. In Japan, the mortality rate of patients with SFTS is approximately 30% [4].

Since SFTSV infection in humans in China was reported in 2009, cases of SFTS have been reported in China, South Korea, and Japan [5–7]. While much progress toward understanding the pathophysiology and clinical characteristics of this disease has been made in recent years [3, 8], limited information is available about the period of viral excretion associated with complications of SFTS. Here, we report a patient with SFTS who developed viremia, encephalopathy and pneumonia and was successively treated with multimodal therapies. The SFTSV genome level was checked in the late stage of the disease in serum, cerebrospinal fluid (CSF), and sputum of the patient.

## Case presentation

### Patient

The patient was a 79-year-old Japanese man who lived in the southwest part of Japan. He had been undergoing hemodialysis for 4 years due to end-stage diabetic nephropathy. He had been working in a bamboo grove surrounding his house for 4 days just before admission (day − 4 to day − 1). Lightheadedness occurred on day − 1, and the next day, he visited a local clinic (day 0). A blood test showed thrombocytopenia [platelet count of 71,000/μL (normal range 158,000-348,000/μL)] and elevated liver enzymes [aspartate transaminase of 287 U/L (normal range 13–30 U/L) and alanine transaminase of 139 U/L (normal range 10–42 U/L)]. Impaired consciousness occurred, and he was then referred to and admitted to Nagasaki University Hospital on the same day (day 0).

### Physical findings on admission day

**Fig. 1:**
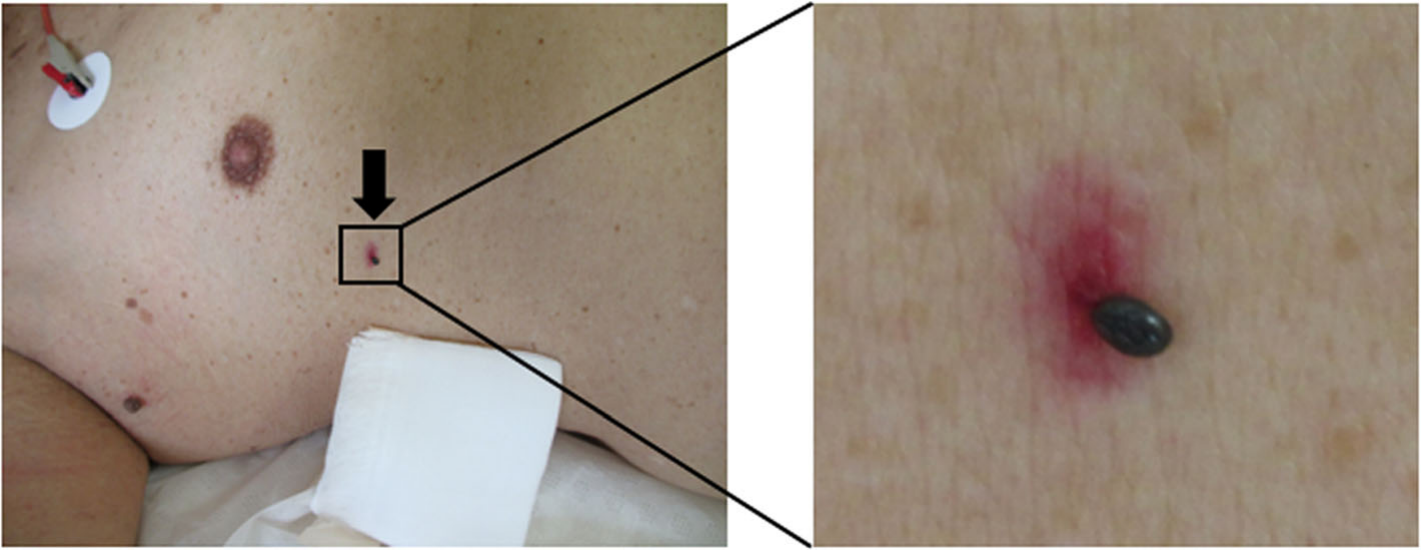
A swollen tick biting the right precordium of the patient (black arrow). SFTSV-RNA was detected from this tick and the patient

On admission (day 0), the patient showed drowsiness with a Japan Coma Scale of II-20 and a Glasgow Coma Scale of 14/15 (E3V5M6). He had a body temperature of 38.4 °C, blood pressure of 141/75 mmHg, pulse rate of 84 beats/min, and respiratory rate of 16 breaths/min. Oxygen saturation was 92% on room air. A swollen tick by blood sucking was attached on the surface of the right precordium (Fig. 1). Palpable cervical and inguinal lymph nodes were found on the right side. He had multiple unraised red spots on the left thigh and erythema associated with exfoliation on the groin of both sides. His chest/abdomen examination did not reveal any abnormal findings. Neck stiffness was not apparent.

### Laboratory and imaging findings on admission day

Blood test results from the first examination are shown in Additional file 1. The hematology test showed pancytopenia, with a white blood cell count of 1800/μL (normal range 3300-8600/μL), hemoglobin of 10.9 g/dL (normal range 13.7–16.8 g/dL), and platelet count of 52,000/μL. Biochemistry tests showed elevation in liver enzymes [aspartate transaminase of 347 U/L, alanine transaminase of 151 U/L, γ-glutamyltransferase of 109 U/L (normal range 13–64 U/L), and lactate dehydrogenase of 878 U/L (normal range 124–222 U/L)]. The serum levels of ferritin and soluble interleukin-2 receptor were 2627 ng/mL (normal range 40–465 ng/mL) and 2135 U/mL (normal range 127–582 U/mL), respectively. Hemophagocytosis was observed in bone marrow aspirates. Blood culture revealed no microorganisms. Right axillary lymphadenopathy was observed in a chest and abdominal radiograph/contrast-enhanced computed tomography (CT) scan. There were no particularly abnormal findings in a noncontrast head CT scan, electrocardiogram, or echocardiograph.

### Clinical course

**Fig. 2:**
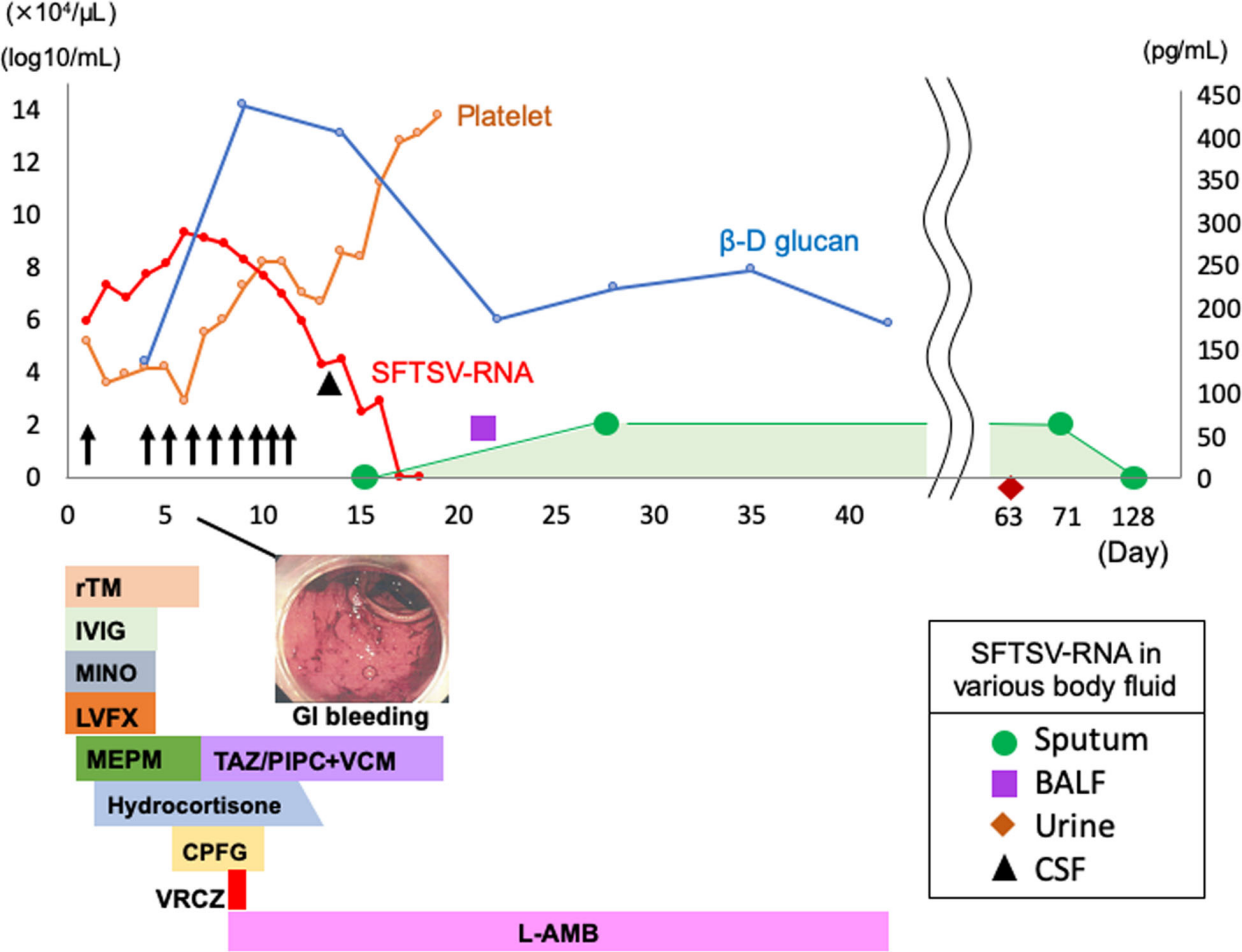
Clinical and microbiological courses of SFTS and invasive fungal infections. The level of SFTSV-RNA peaked on day 5 of admission and became undetectable (below the detection limit) on day 16. As the level of SFTSV-RNA decreased, platelet counts recovered without platelet transfusion (arrows). Sputum SFTSV-RNA was positive until day 71 even after serum SFTSV-RNA became undetectable. Sputum SFTSV-RNA became undetectable on day 128. SFTSV, severe fever with thrombocytopenia syndrome virus; rTM, recombinant human thrombomodulin; IVIG, intravenous immunoglobulin; MINO, minocycline; LVFX, levofloxacin; MEPM, meropenem; TAZ/PIPC, tazobactam/piperacillin; VCM, vancomycin; CPFG, caspofungin; VRCZ voriconazole; L-AMB, liposomal amphotericin B; BALF, bronchoalveolar lavage fluid; CSF, cerebrospinal fluid

On admission (day 0), differential diagnoses included SFTS and rickettsial infections. In accordance with the treatment for severe rickettsial infections, we started infusion of minocycline (200 mg/day) and levofloxacin (500 mg/day) combined with platelet transfusion and recombinant human thrombomodulin administration (8320 U/day) (Fig. 2). Intravenous immunoglobulin was given as an adjunct therapy. On day 1, unconsciousness rapidly progressed, and a generalized seizure with respiratory failure occurred. His respiration was supported with mechanical ventilation, and systemic management, including continuous hemodiafiltration, was initiated in an intensive care unit. Administration of meropenem (3 g/day) was also started. On day 2, a real-time quantitative reverse transcription-polymerase chain reaction (qRT-PCR) assay for SFTSV, which was performed as described previously [9], was positive (5.97 log_10_ copies/mL) in the serum. Furthermore, qRT-PCR for the tick that had bitten the patient was positive (see Additional file 2). No Rickettsiaceae, including *Orientia tsutsugamushi* and *Rickettsia japonica,* was detected by duplex real-time PCR [10] using blood and eschar samples.

**Fig. 3:**
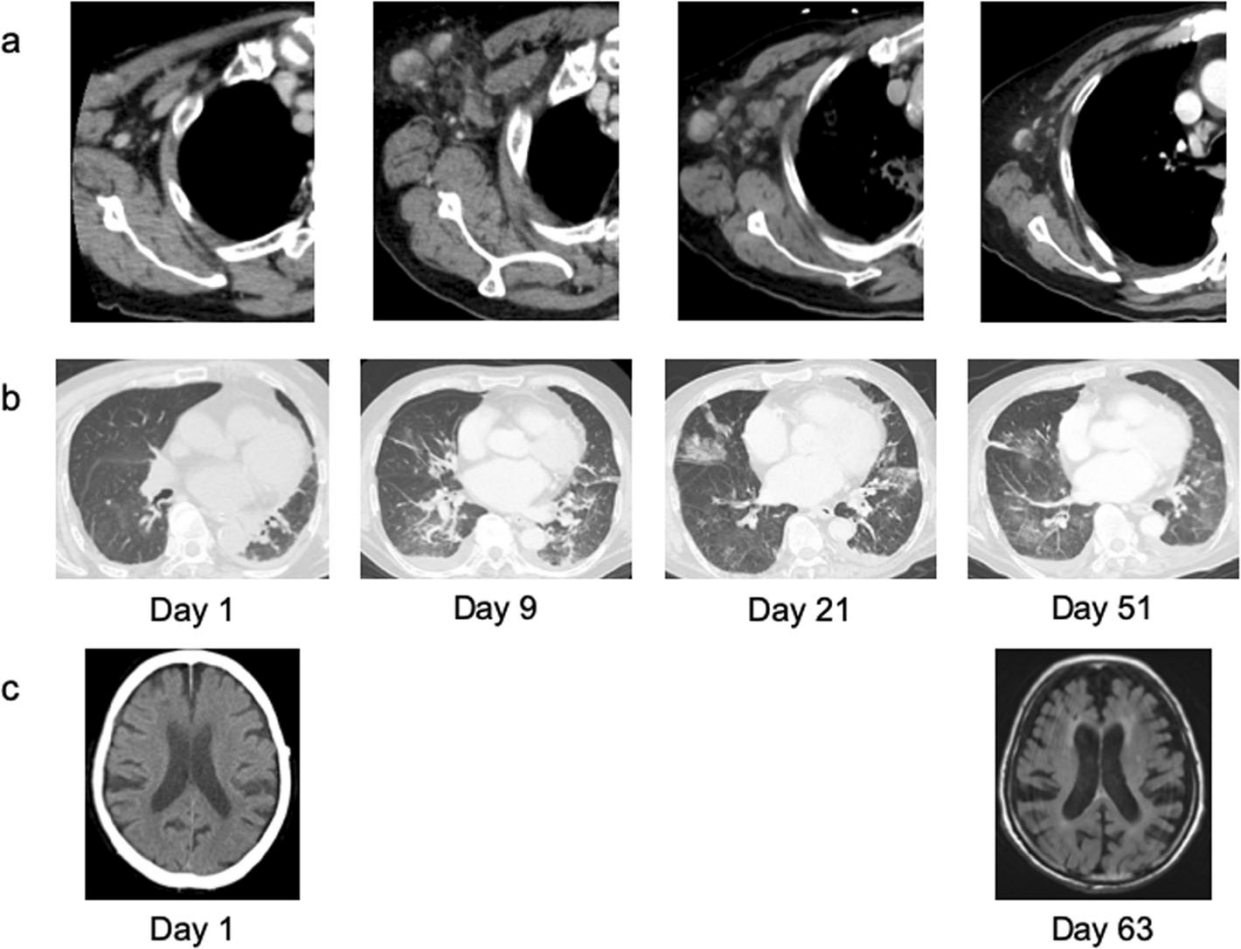
Chest and brain imaging. (a) Right axillary lymphadenopathy gradually reduced. (b) Chest CT showed diffuse ground glass opacity on days 9, 21, and 51. (c) Head CT (Day 1) and MRI (Day 63) showed no remarkable findings except cerebral atrophy

We administered 200 mg/day hydrocortisone from day 2 considering the possibility of relative adrenal insufficiency. The levels of liver enzymes significantly increased on day 4, and minocycline, levofloxacin and immunoglobulin preparations were discontinued. Because β-D glucan levels increased at 131.1 pg/mL (MK-II assay; negative cutoff value < 20 pg/mL) and both serum *Aspergillus* and *Candida* antigen were positive with a value of 4.6 for *Aspergillus* antigen (negative cutoff index < 0.5), the central venous catheter was replaced, and presumptive therapy with caspofungin 70 mg/day was initiated (Fig. 2). After that, serum *Cryptococcus* antigen was positive (titer; 1:1), and considering the possibility of trichosporonosis, voriconazole 280 mg/day was added to the treatment regimen. On day 5, the serum SFTSV-RNA level reached a peak (9.31 log_10_ copies/mL) and then decreased. On day 6, severe melena appeared. Lower gastrointestinal endoscopy showed mucous and bloody stool, intestinal edema, and severe rectal hemorrhage. A stool culture detected *Candida glabrata*. On day 8, β-D glucan levels further increased to 425.5 pg/mL, and a blood culture detected *Candida glabrata*; therefore, we judged that bacterial translocation occurred from the intestine. Caspofungin and voriconazole were changed to liposomal amphotericin B 250 mg/day, and meropenem was changed to the combination therapy with tazobactam/piperacillin and vancomycin. The levels of liver enzymes peaked on day 8, after which they tended to decrease. After day 12, platelet transfusion became unnecessary. Because impaired consciousness continued even after the sedative was discontinued, encephalopathy was suspected. A lumbar puncture was performed on day 13, and CSF was obtained. The qRT-PCR of CSF to detect SFTSV was positive with a value of 4.10 log_10_ copies/mL. On day 16, the serum SFTSV-RNA level dropped below the detectable level. On day 31, the patient was discharged from the intensive care unit, and the level of consciousness improved approximately one month after disease onset. There was a swollen right axillary lymph node at the first examination, which remained swollen after viruses disappeared from the blood (Fig. 3). However, we could not judge whether SFTSV remained in the lymph node because we did not perform a lymph node biopsy.

Although a chest CT scan on admission did not show clear pneumonia findings, infiltrative shadows on the basal area of both lungs were found by a chest CT scan on day 9 (Fig. 3). In bronchoalveolar lavage fluid (BALF) obtained on day 21, neutrophils were predominant in the cellular fraction, but general types of bacteria were not detected, probably because broad-spectrum antimicrobials had already been administered. However, *Cryptococcus* antigen (titer; 1:2), *Aspergillus* antigen (a value of 8.6), and SFTSV-RNA (2.51 log_10_ copies/mL) were detected, and *Aspergillus niger* was cultured from the BALF. Therefore, we speculated that fungal pneumonia was induced by transient immunosuppression caused by SFTSV infection and continued liposomal amphotericin B administration. A chest CT scan on day 30 showed that infiltrative shadows were organized overall. Liposomal amphotericin B and tazobactam/piperacillin were discontinued on days 37 and 42, respectively. A slight ground-glass opacity remained, but infiltrative shadows improved on day 51. Although β-D glucan levels remained high and *Aspergillus* antigen was persistently positive, there was no relapse of aspergillosis or candidemia. SFTSV-RNA was persistently detected in the sputum until day 71 despite its disappearance in the blood on day 16. After confirming that SFTSV-RNA became negative in the sputum on day 127, we stopped performing droplet isolation precautions on the patient.

## Discussion and conclusions

A meta-analysis on risk factors for mortality in patients with SFTS has shown advanced age; high viremia levels; low albumin levels; low platelet count; high aspartate transaminase, alanine transaminase, lactate dehydrogenase, and creatine kinase levels; and prolonged activated partial thromboplastin time [11]. The patient met all of the above factors, and his disease state was severe.

Patients with SFTS are prone to develop encephalopathy [12, 13]. Cui et al. reported that 19% of patients with SFTS developed encephalopathy, and of these, the mortality rate was 44.7% [12]. In their study, early symptoms mainly included dimness of vision, bad feeling, cramp, sleepiness, and coma, which occurred within approximately 5 days after disease onset. Most risk factors for encephalopathy were consistent with the aforementioned factors related to poor prognosis. In addition, Park et al. reported that CSF analysis exhibited glucose and protein levels within the normal range, an increase in cell count in the CSF was not remarkable, and the SFTSV genome was positive in 75% of cases [13]. These findings, along with the findings in this study, may be an important piece of information to advance our understanding of the pathophysiological mechanisms of encephalopathy in SFTS patients. Encephalopathy/encephalitis is a common complication of SFTS, and SFTSV-RNA is frequently detected, especially in the CSF of patients with symptoms or signs of central nervous system manifestations [13]. Although a positive PCR result for the SFTSV genome does not necessarily indicate infectivity of the virus unless culture isolation of live virus is not demonstrated, strict contact isolation precautions should be considered when performing CSF study in SFTS patients regardless of serum viral load.

In previous reports, SFTSV was detected not only in the blood and CSF but also in the urine, gastric juice, sputum, and semen [9, 14]. A case in which viruses did not disappear after plasma exchange has also been reported [14]. Moreover, there were several case reports that strongly suggest droplet infection [15], including a case in which a patient with SFTS developed severe pneumonia in an early stage after disease onset, and two caregiving family members also developed SFTS [16]. In addition, there was a case report of SFTS development in two persons who did not touch the body fluid of an SFTSV-infected corpse but stayed for a long period of time in a small room where the corpse was laid, indicating a possibility of aerosol transmission of SFTSV [17]. In the present case, we isolated the patient in a negative-pressure airborne-isolation room from the time of the first examination and managed with droplet/contact isolation precautions. Specifically, we wore N95 masks, aprons, gloves, and face shields. In the intensive care unit, isolation with curtains and zoning were maintained to promote precaution. Although blood virus levels became undetectable on day 16, the virus genome had been persistently positive in sputum, and thus, the same countermeasures against infection were continuously conducted. Virus genomes in sputum remained positive until day 79. However, we confirmed the negativity on day 127 and subsequently removed countermeasures against droplet infection. Taken together, there might be cases in which SFTS virus might remain in various fluids and tissues even after it disappears from the blood. It may be possible that body fluids such as respiratory excretions become a source of infection; thus, careful management is needed.

In conclusion, we herein present an SFTS patient in whom encephalopathy, pneumonia, and invasive fungal infections developed. SFTSV-RNA was detected in the patient and the tick that bit this patient. Although the general condition improved and serum SFTSV levels reached a peak and decreased, a high level of SFTSV-RNA was detected in the CSF. Furthermore, the virus genome remained positive in the sputum for a long period of time, although viremia and pneumonia improved. These findings indicate that even when blood virus levels become undetectable, SFTSV may remain in other body fluids or tissues. Body fluids such as sputum can be a potential source of infection to others; thus, careful attention is necessary for countermeasures against SFTSV infection.

## Supplementary information

**Additional file 1.** Table 1. Laboratory data from the first examination

**Additional file 2.** Detection of SFTSV-RNA from a tick with qRT-PCR. Materials and methods for qRT-PCR and a figure showing amplification curves.

## Data Availability

The datasets used and/or analysed during the current study are available from the corresponding author on reasonable request.

## Abbreviations

BALF: Bronchoalveolar lavage fluid
CSF: Cerebrospinal fluid
CT: Computed tomography
PCR: Polymerase chain reaction
SFTS: Severe fever with thrombocytopenia syndrome
SFTSV: Severe fever with thrombocytopenia syndrome virus
