# Supplementary material for: Detection of viral RNA in diverse body fluids in an SFTS patient with encephalopathy, gastrointestinal bleeding and pneumonia: a case report and literature review
Source: BMC Infect Dis. 2020 Apr 15;20:281. doi: 10.1186/s12879-020-05012-8 (PMC7160946; doi:10.1186/s12879-020-05012-8)
Supplement: Supplementary file 1 — Additional file 1. Table 1. Laboratory data from the first examination [file 12879_2020_5012_MOESM1_ESM.docx]

**Table 1. Laboratory data** **from the first examination**

| **Blood examination** | | | | | |
| --- | --- | --- | --- | --- | --- |
| **Hematology** | |  | **Biochemistry** | |  |
| White blood cell | 1.8 (3.3-8.6) | × 10^3^/μL | Na | 136 (138-145) | mEq/L |
| Segmented neutrophil | 77.2 (38.0-74.0) | % | K | 5.1 (3.6-4.8) | mEq/L |
| Lymphocyte | 17.1 (16.5-49.5) | % | Cl | 100 (101-108) | mEq/L |
| Monocyte | 5.7 (2.0-10.0) | % | cCa | 9.3 (8.8-10.1) | mg/dL |
| Eosinophil | 0 (0.0-8.5) | % | BUN | 43 (8-20) | mg/dL |
| Basophil | 0 (0.0-2.5) | % | Cr | 11.1 (0.95-1.07) | mg/dL |
| Red blood cell | 3.77 (4.35-5.55) | × 10^6^/μL | TP | 7.2 (6.6-8.1) | g/dL |
| Hemoglobin | 10.9 (13.7-16.8) | g/dL | Alb | 3.5 (4.1-5.1) | g/dL |
| Hematocrit | 32.6 (40.7-50.1) | % | TB | 0.7 (0.4-1.5) | mg/dL |
| Platelet | 5.2 (15.8-34.8) | × 10^4^/μL | AST | 347 (13-30) | U/L |
|  |  |  | ALT | 151 (10-42) | U/L |
| **Coagulation** |  |  | ALP | 258 (106-322) | U/L |
| PT | 94 (70-130) | % | γ-GT | 109 (13-64) | U/L |
| PT-INR | 1.03 |  | LDH | 878 (124-222) | U/L |
| APTT | 54.7 (25-38) | s | ChE | 235 (240-486) | U/L |
| ATIII | 59 (75-125) | % | TG | 215 (40-234) | mg/dL |
| FDP | 55.9 (<5.0) | μg/mL | CK | 143 (59-248) | U/L |
| D-dimer | 29.6 (<1.0) | μg/mL | Amylase | 303 (44-132) | U/L |
|  |  |  | CRP | 4.3 (0.00-0.14) | mg/dL |
| **Infection** |  |  | PCT | 1.4 (≦0.05) | ng/mL |
| SFTSV-RNA (Serum) | 5.97 log_10_ | copies/mL | Glu | 144 (73-109) | mg/dL |
| β-D glucan | 131.3 | pg/mL | HbA1c | 6.4 (4.9-6.0) | % |
| *Aspergillus* Ag | 4.6 |  | NT-proBNP | 8468 (≦55) | pg/mL |
| *Cryptococcus* Ag | 1+ |  | IgG | 1076 (861-1047) | mg/dL |
| *Candida* Ag | + |  | Ferritin | 2627 (40-465) | ng/mL |
| C7-HRP | − |  | sIL-2R | 2135 (127-582) | U/mL |
| **Cerebrospinal fluid** | | | | | |
| Total cell counts | 6 (0-5) | /mm^3^ | *Cryptococcus* Ag | - |  |
| Mononuclear cell | 83 | % | SFTSV-RNA | 4.1 log_10_ | copies/mL |
| Protein | 130 | mg/dL |  |  |  |
| Glucose | 83 | mg/dL |  |  |  |
| **Bronchoalveolar lavage fluid** | | | | | |
| Total cell counts | 6.0 × 10^5^ | /mL | Diff quik stain | - |  |
| Macrophage | 44 | % | Grocott stain | - |  |
| Neutrophil | 43 | % | Bacteria | - |  |
| Basophil | 1 | % | Acid-fast bacillus | - |  |
| Lymphocyte | 12 | % | CMV-DNA | + |  |
| Eosinophil | 0 | % | *Pneumocystis* DNA | - |  |
| CD4/8 ratio | 0.3 |  | *Cryptococcus* Ag | 2+ |  |
|  |  |  | *Aspergillus* Ag | 8.558 |  |
|  |  |  | SFTSV-RNA | 2.51 log_10_ | copies/mL |

Normal ranges are given in parentheses.

*PT,* prothrombin time; *PT-INR,* prothrombin time-international normalized ratio; *APTT,* activated partial thromboplastin time; *ATIII*, antithrombin III; *FDP,* fibrinogen degradation products; *Na,* sodium; *K*, potassium; *Cl,* chlorine; *cCa* corrected calcium, *BUN,* blood urea nitrogen; *Cr,* creatinine; *TP,* total protein; *Alb* albumin; *TB,* total bilirubin; *AST,* aspartic aminotransferase; *ALT,* alanine aminotransferase; *ALP,* alkaline phosphatase; *γ-GT,* γ-glutamyltransferase; *LDH,* lactate dehydrogenase; *ChE*, choline esterase; *TG*, triglyceride; *CK,* creatine kinase; *CRP,* C-reactive protein; *PCT*, procalcitonin; *Glu,* glucose; *HbA1c,* hemoglobin A1c; *sIL-2R*, soluble interleukin-2 receptor; *NT-proBNP*, N-terminal pro-brain natriuretic peptide; *IgG,* immunoglobulin G; *Ag,* antigen; *SFTSV*, severe fever with thrombocytopenia syndrome virus; *CMV*, cytomegalovirus.
