# Supplementary material for: Detection of viral RNA in diverse body fluids in an SFTS patient with encephalopathy, gastrointestinal bleeding and pneumonia: a case report and literature review
Source: BMC Infect Dis. 2020 Apr 15;20:281. doi: 10.1186/s12879-020-05012-8 (PMC7160946; doi:10.1186/s12879-020-05012-8)

**Detection of SFTSV-RNA from a tick with qRT-PCR**

A tick removed from the patient was put into a 2 mL tube containing 1 mL of ISOGEN II (Nippongene, Tokyo, Japan), 6.35 mm ceramic spheres (MP Biomedicals, Illkirch, France), and garnet matrix (MP Biomedicals). After the sample was shaken for 30 seconds (sec) at 4,200 rpm using a Mini Bead Beater (BioSpec Products, Inc., Bartlesville, OK), total RNA was extracted from the tick using ISOGEN II (Nippongene) and p-bromoanisole (Wako, Osaka, Japan) according to the manufacturer’s instructions. The resulting RNA pellet was dissolved in 50 µL of DEPC-treated water (Nippongene). The specific primers (5’- GCA ACA AGA TCG TCA AGG CAT C-3’ and 5’-TGC TGCA GCA CAT GTC CAA GTG G-3’) and Taqman MGB probe (5’-FAM-CTG GTT GAG AGG GCA-MGB-NFQ-3’) targeting the SFTS Small (S) segment were designed using Primer Express 3 software (Applied Biosystems, Foster City, CA, USA). qRT-PCR was performed in a 50 µL reaction mixture containing 1× RNA-direct Real-time PCR Master Mix, 2.5 mM Mn(OAc)_2_, 0.32 µM each primer, 0.16 µM MGB probe, and 5 µL of total RNA on the LightCycler 480 Instrument II (Roche Diagnostics GmbH, Mannheim, Germany). The reaction mixture supplied with the tenfold serial dilution of the control plasmid (1×10^7^ to 1×10^1^ copies per reaction) was also prepared. The reaction conditions were as follows: initial denaturation at 90°C for 30 sec; reverse transcription at 61°C for 20 min; denaturation at 95°C for 30 sec; 50 cycles of denaturation at 95°C for 5 sec, annealing at 64°C for 60 sec for amplification with quantification. Viral copy number per reaction was calculated using Roche LightCycler software version 1.5 analyzed with the Abs Quant/Fit Points method with the default setting.

The amplification curve of the sample obtained from the tick (Figure, red line).


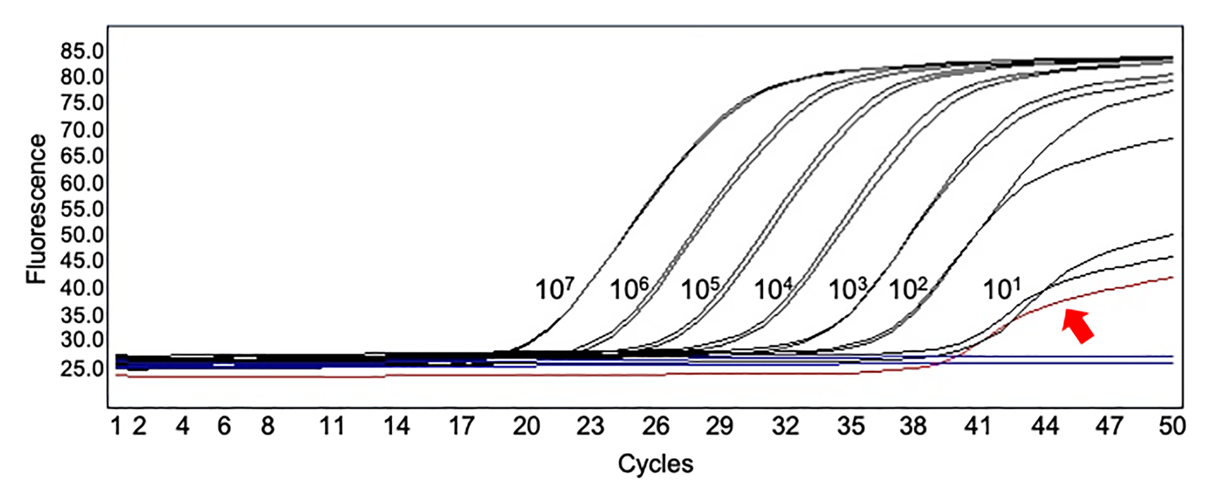

Supplement: Supplementary file 2 — Additional file 2. Detection of SFTSV-RNA from a tick with qRT-PCR. Materials and methods for qRT-PCR and a figure showing amplification curves. [file 12879_2020_5012_MOESM2_ESM.docx]
